# Acute flaccid paralysis (AFP) surveillance: challenges and opportunities from 18 years’ experience, Spain, 1998 to 2015

**DOI:** 10.2807/1560-7917.ES.2018.23.47.1700423

**Published:** 2018-11-22

**Authors:** Josefa Masa-Calles, Nuria Torner, Noemí López-Perea, María de Viarce Torres de Mier, Beatriz Fernández-Martínez, María Cabrerizo, Virtudes Gallardo-García, Carmen Malo, Mario Margolles, Margarita Portell, Natividad Abadía, Aniceto Blasco, Sara García-Hernández, Henar Marcos, Núria Rabella, Celia Marín, Amelia Fuentes, Isabel Losada, Juan García Gutiérrez, Alba Nieto, Visitación García Ortúzar, Manuel García Cenoz, José María Arteagoitia, Ángela Blanco Martínez, Ana Rivas, Daniel Castrillejo, Spanish AFP Surveillance Working Group

**Affiliations:** 1 National Centre for Epidemiology, Instituto de Salud Carlos III, Madrid, Spain; 2 Spanish Consortium for Research in Epidemiology and Public Health (CIBERESP), Instituto de Salud Carlos III, Madrid, Spain; 3 Public Health Agency of Catalonia, Generalitat of Catalonia, Spain; 4 Department of Medicine, University of Barcelona, Barcelona, Spain; 5 National Polio Laboratory, National Centre for Microbiology, Instituto de Salud Carlos III, Madrid, Spain; 6 Servicio de Vigilancia y Salud Laboral, Consejería de Salud, Andalucía, Spain; 7 Servicio de Vigilancia en Salud Pública, D.G. de Salud Pública, Departamento de Sanidad, Aragón, Spain; 8 Servicio de Vigilancia Epidemiológica, Consejería de Sanidad, Instituto de Investigación Sanitaria del Principado de Asturias, Spain; 9 Servicio de Vigilancia Epidemiológica, Conselleria de Salut, Família i Bienestar Social Baleares, Spain; 10 Servicio de Epidemiologia y Prevención, Dirección General Salud Pública, Servicio Canario de Salud, Canarias, Spain; 11 Sección de Vigilancia Epidemiológica, D.G. de Salud Pública, Cantabria, Spain; 12 Servicio de Epidemiología, D.G. de Salud Pública, Consejería de Sanidad, Castilla-La Mancha, Spain; 13 Servicio de Vigilancia Epidemiológica y Enfermedades Transmisibles, D.G. de Salud Pública, Consejería de Sanidad, Castilla y León, Spain; 14 Microbiology Department, Hospital de la Santa Creu i Sant Pau, Microbiology & Genetics Department, Universitat Autònoma de Barcelona, Barcelona, Spain; 15 Servei de Vigilància i Control Epidemiològic, Conselleria de Sanitat Universal i Salut Pública, Comunitat Valenciana, Spain; 16 Subdirección de Epidemiología. D.G. de Salud Pública, Servicio Extremeño de Salud, Consejería de Salud y Políticas Sociales, Extremadura, Spain; 17 Servizo de Epidemioloxía, Dirección Xeral de Saúde Pública, Consellería de Sanidade, Xunta de Galicia, Spain; 18 Servicio de Epidemiología, Subdirección General de Epidemiología, Dirección General de Salud Pública, Comunidad Autónoma de Madrid, Spain; 19 Servicio de Epidemiología, D. G. de Salud Pública y Adicciones, Consejería de Salud, Región de Murcia, Spain; 20 Servicio de Epidemiología y Prevención Sanitaria, Instituto de Salud Pública de Navarra, IdiSNA, Spain; 21 Servicio de vigilancia epidemiológica y vacunas, Dirección de Salud Pública y Adicciones, Departamento de Salud, País Vasco, Spain; 22 Sección de Vigilancia Epidemiológica y Control de Enfermedades Transmisibles, D.G de Salud Pública y Consumo, Consejería de Salud, La Rioja, Spain; 23 Servicio de Epidemiología, Consejería de Sanidad y Consumo, Ceuta, Spain; 24 Servicio de Epidemiología, D.G. de Sanidad y Consumo, Consejería de Presidencia y Salud Pública, Melilla, Spain; 25 The other members of the Spanish AFP Surveillance Working Group are listed at the end of this article

**Keywords:** acute flaccid paralysis surveillance, poliomyelitis eradication, enterovirus, surveillance, Spain, vaccine preventable disease

## Abstract

Acute flaccid paralysis (AFP) surveillance is key for global polio eradication. It allows detecting poliovirus (PV) reintroductions from endemic countries. This study describes AFP surveillance in Spain from 1998 to 2015. During this time, 678 AFP cases were reported to the Spanish National Surveillance Network. The mean notification rate was 0.58 AFP cases/100,000 population under 15 years old (range: 0.45/100,000–0.78/100,000). Two periods (P) are described: P1 (1998–2006) with the AFP notification rate ranging from 0.66/100,000 to 0.78/100,000, peaking in 2001 (0.84/100,000); and P2 (2007–2015) when the AFP rate ranged from 0.43/100,000 to 0.57/100,000, with the lowest rate in 2009 (0.31/100,000). No poliomyelitis cases were caused by wild PV infections, although two Sabin-like PVs and one imported vaccine-derived PV-2 were detected. Overall, 23 (3.4%) cases met the hot case definition. Most cases were clinically diagnosed with Guillain–Barré syndrome (76.9%; 504/655). The adequate stool collection rate ranged from 33.3% (7/21) to 72.5% (29/40). The annual proportion of AFP cases with non-polio enterovirus findings varied widely across the study period. AFP surveillance with laboratory testing for non-polio enteroviruses must be maintained and enhanced both to monitor polio eradication and to establish sensitive surveillance for prompt detection of other enteroviruses causing serious symptoms.

## Introduction

The 1988 Global Polio Eradication Initiative (GPEI) included four strategies: (i) high systematic immunisation with oral polio vaccine (OPV); (ii) provision of supplementary vaccinations; (iii) active surveillance of wild poliovirus (WPV) through reporting and laboratory testing of all acute flaccid paralysis (AFP) cases in children < 15 years of age; (iv) once transmission has been limited, development of campaigns to reach any unvaccinated population [1].

By 2011, four of the six World Health Organization (WHO) Regions had seen their last indigenous polio cases and were certified as polio free. But polio proved to be insidious and poliovirus transmission has persisted in countries with ineffective immunisation systems and low-performing eradication activities, resulting in potential risk of international spread and virus importations into previously polio-free countries [2].

Despite the dramatic decrease in worldwide polio cases in the last few decades, the WHO estimates that, from 2003 to 2014, there were 191 importation events into previously polio-free countries, resulting in 3,763 reported cases of paralytic polio in 43 countries. A Public Health Emergency of International Concern was declared by the WHO in 2014 [3]. Since then the International Health Regulations Committee recommends that countries in which WPV or vaccine-derived poliovirus (VDPV) transmission is occurring should ensure and document that residents and long-term visitors (i.e. > four weeks) receive a dose of polio vaccine prior to international travel. Restriction of international travel of any resident lacking documentation of appropriate polio vaccination should be imposed at the point of departure [3].

On rare occasions, vaccine-associated paralytic poliomyelitis (VAPP) may occur up to 30 days after receiving an OPV dose. Sabin polioviruses can also replicate in immunocompromised persons (iVDPV) acquiring the neurovirulence and transmissibility characteristics of WPV and they can spread in populations with low OPV coverage causing circulating VDPV (cVDPV) cases and outbreaks [4]

In September 2015, worldwide eradication of indigenous WPV type-2 was declared. To stop circulation of VDPV type-2, live attenuated type-2 poliovirus was withdrawn from oral vaccines as at April 2016. Nevertheless outbreaks caused by cVDPVs, including cVDPV type-2, have continued in areas of several countries [5]. This implies that long-term use of OPV poses an ongoing risk and, consequently the GPEI will need to stop OPV vaccination in addition to certification and WPV containment [5]. 

The European Regional Commission for Poliomyelitis Eradication (RCC) publishes an annual report with the main features of national reports and conclusions about the polio-free status of the Region. The possibility of re-entry of the virus into Europe can never be completely discarded primarily because of low population immunity in some countries [6].

The National Certification Committee (NCC) for polio eradication was established in Spain in 1998 as a component of the National Plan of Actions Aimed at the Achievement of the Certificate of Polio Eradication (NPCPE) [7]. An annual update on polio eradication activities is prepared by the NCC and submitted to the RCC. The report contains a statement presenting the evidence-based rationale for the absence of poliovirus circulation in the country.

Updated versions of the NPCPE were approved in Spain in 2007, 2011 and 2016. Nationwide, the main poliovirus eradication activities are: maintenance of high vaccination coverage and the strengthening of poliovirus surveillance, including AFP and enterovirus (EV) surveillance [7-9].

**Figure 1:**
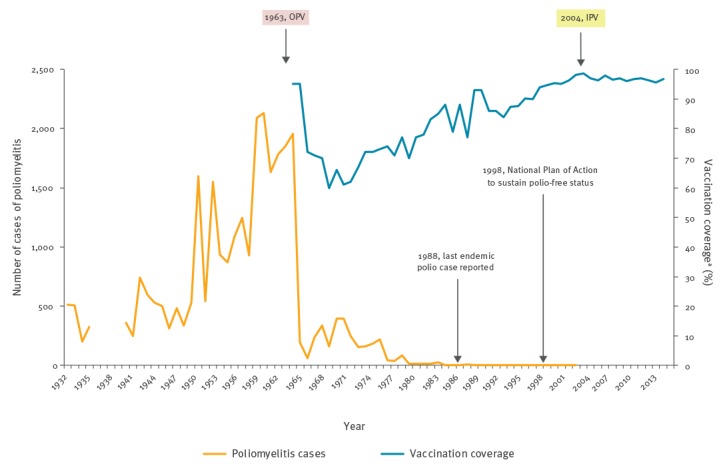
Cases of poliomyelitis, type of polio vaccines and vaccination coverage, Spain, 1931–2015

IPV: inactivated polio vaccine; OPV: oral polio vaccine.

^a^ Vaccination coverage with three doses of polio vaccine by 12 months of age.

Source: National Centre of Epidemiology, Instituto de Salud Carlos III and Ministry of Health.

Since the implementation of the NPCPE and the development of the AFP surveillance system, the sensitivity of the surveillance has gradually decreased in relation to the WHO objective of being able to detect at least one case of AFP per 100,000 population under 15 years old [9]. In Spain, OPV was replaced by inactivated polio vaccine (IPV) in 2004. Since 1996, national coverage of three doses in children’s first year of age exceeds 95%. In 2015, national coverage was 96.4% (ranging between 92.8 and 100.0% according to autonomous regions) (Figure 1).

The objective of this study is to report and evaluate the results of 18 years (1998–2015) of the surveillance programme for AFP in Spain in terms of incidence, epidemiological and laboratory investigations, clinical diagnosis, as well as utility and quality of surveillance.

## Methods

### Acute flaccid paralysis surveillance system in Spain

In Spain, AFP surveillance is the main component of poliovirus surveillance, along with supplementary EV laboratory surveillance. Environmental surveillance is not established nationwide. Laboratory techniques and protocols that should be maintained or could be developed in the event of PV importation or an outbreak are assessed annually by a pilot environmental study in an urban area. The results are reported yearly to the RCC.

**Figure 2:**
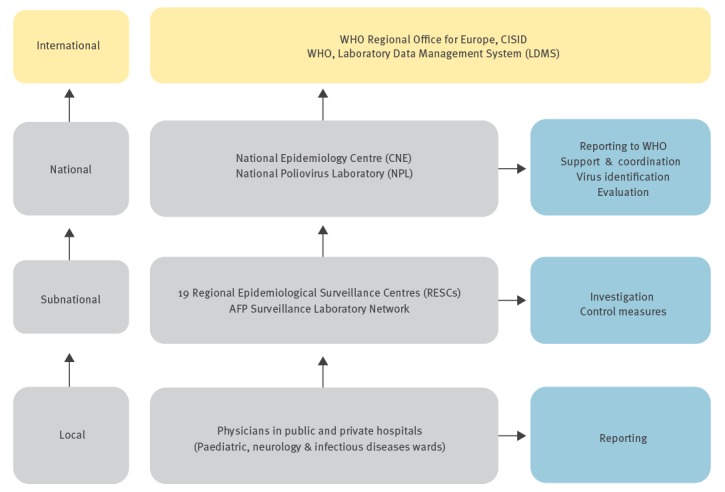
Acute flaccid paralysis surveillance system in Spain, reporting information flowchart

AFP: acute flaccid paralysis; CISID: Centralized Information System for Infectious Diseases; WHO: World Health Organization.

AFP surveillance is mandatory in Spain. It is conducted by the Spanish Epidemiological Surveillance System (RENAVE) together with the AFP Surveillance Laboratory Network, and is coordinated by the National Epidemiology Centre (CNE) of the Carlos III Health Institute (ISCIII). The Regional Epidemiological Surveillance Centres (RESCs) are located in each of the 19 autonomous territories (17 autonomous regions and two autonomous cities). These surveillance centres are made up of local networks of public and private hospitals (paediatric, neurology and infectious disease wards) which are directly coordinated by the person in charge of the RESCs (Figure 2).

Case notification as well as other relevant information is collated centrally and subsequently fed-back through the system, between the different levels: local, regional, national and international (Figure 2).

### Laboratory network

The AFP Surveillance Laboratory Network coordinated by the National Poliovirus Laboratory (NPL) of the ISCIII was set up in 1998. The NPL is part of the European Polio Laboratory Network and is accredited annually by the WHO. In Spain the number of regional laboratories belonging to the AFP surveillance network has been reduced over time, decreasing from nine in 1998 to only three in 2015. The network laboratories perform virological studies of notified AFP cases in their region, while the national laboratory studies all AFP cases reported by the 16 autonomous territories without regional laboratories.

The network laboratories perform virological studies of stool samples using cell culture techniques – following protocols recommended by the WHO [10,11]. Since 2015 a new diagnosis policy has been introduced in all European polio laboratories and now culture results must be available within 14 days after laboratories receive samples, instead of 28 days earlier [9]. Laboratory analysis consists of the evaluation of viral growth after two blind passages on cell lines (RD and L20B) sensitive to poliovirus infection (3 serotypes) and to most of the non-polio EV (NPEV) infections. Negative results refer to the absence of viral growth. Any positive result must be sent to the national laboratory immediately. The NPL is responsible for confirming EV detection and characterising the serotype. Any virological results will be notified to the WHO through the Laboratory Data Management System (LDMS) (Figure 2).

### Reporting and investigation of acute flaccid paralysis cases

**Figure 3:**
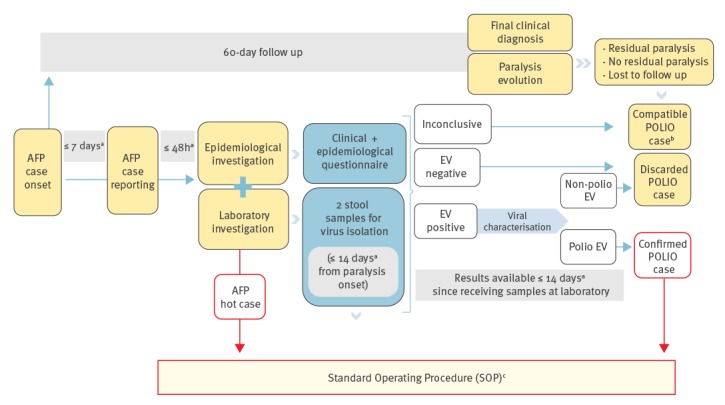
Acute flaccid paralysis surveillance system in Spain, case reporting, investigation, classification and follow-up, Spain, 1998–2015

AFP: acute flaccid paralysis; EV: enterovirus.

^a^ Maximum accepted time delay intervals for assuring the surveillance quality.

^b^ A 60-day follow-up with clinical revision must be performed for compatible polio cases.

^c^ Standard operating procedures include priority for laboratory investigation, case and contact investigation, assessment of population immunity and enhanced surveillance.

The RESCs must notify the CNE of any AFP cases occurring in the 0–14 year-old population. In the event of an AFP case, a preliminary reporting form with clinical and epidemiological information has to be completed [12]. A hot case is a person highly suspected for polio based on the clinical suspicion of poliomyelitis, and who has either received less than three doses of polio vaccine, or has recently travelled to an endemic country (having returned up to 35 days before the onset of paralysis) or belongs to a high risk group [13]. If an AFP hot case is reported, a set of Standard Operating Procedures (SOP), including priority for laboratory investigation and contact tracing, must be implemented [9] (Figure 3).

Clinical specimens for virological investigation have to be collected. A follow-up reporting form is sent to CNE to determine paralysis evolution after 60 days from paralysis onset, and to clarify the final diagnosis of the case. Cases are classified as confirmed, discarded or compatible with polio [12,13] (Figure 3).

### Zero reporting

To monitor AFP surveillance quality zero reporting is recommended. The collaborating hospitals in the region should respond to RESC enquiries through a contact-point among the medical staff, who acts as a link between clinicians and epidemiologists for surveillance activities. To maintain the highest coverage, a number of hospitals report for each region*:* Andalucía (n = 33); Aragón (n = 9); Asturias (n = 1); Baleares (n = 14); Cantabria (n = 1); Canarias (n = 4); Castilla La Mancha (n = 12); Castilla y León (n = 18); Cataluña (n = 14); Comunidad Valenciana (n = 28); Extremadura (n = 8); Galicia (n = 10); Madrid (n = 18); Murcia (n = 10); Navarra (n = 1); País Vasco (n = 6); La Rioja (n = 2); Ceuta (n = 1) and Melilla (n = 1)*.* On a monthly basis, medical staff are asked to report non-notified AFP cases in hospitalised children. If AFP cases are not found, a zero-reporting form should be sent to the CNE in the first 7 days of the following month. The completeness and the timeliness of zero reporting are used as surveillance quality indicators (see Table 1 in the results’ section).

### Assessing acute flaccid paralysis surveillance by reviewing hospital discharge registries

Retrospective searches of medical records which meet AFP clinical criteria help to determine a baseline burden for AFP in a country. Annually, each RESC conducts a retrospective search at the Regional Hospitalization Discharge Registry. The results of these searches are reported to the CNE. The cause of hospitalisation is considered to be clinically compatible with AFP when the Ninth International Classification of Diseases (ICD-9-CM) [14] codes are 357.0 and 356.9 (Guillain–Barré Syndrome or other peripheral neuropathy), 336.9 (acute myelitis) or 045.0 and 045.1 (acute poliomyelitis caused by poliovirus or other neurotropic viruses).

### Assessment of quality of acute flaccid paralysis surveillance performance

A set of indicators are calculated annually at national level and compared with WHO standards. Indicators evaluate the main system attributes: sensitivity, completeness and timeliness of notification, investigation and laboratory workup. To synthetise the quality of AFP surveillance WHO requests the ‘surveillance index’ (see Table 1 in the results’ section).

### Data analysis

Individualised information regarding notified AFP cases between 1998 and 2015 in Spain was obtained from the RENAVE database. Zero-case monthly reports and data from hospital discharge registries between 2000 and 2015 were obtained from the AFP surveillance system. To calculate the AFP notification rate, population data were obtained from the National Institute for Statistics.

## Results

### Acute flaccid paralysis detection and demographic variables

A total of 678 AFP cases were notified between 1998 and 2015. The annual number of AFP cases reported ranged from 51 (in 2001) to 21 (in 2009) respectively. All of the cases were discarded for polio except two VAPPs (1999; 2001) and one paralysis case associated with an iVDPV (2005). The AFP detection rate in the population under 15 years decreased over the study period, from 0.78 AFP cases/100,000 in 1998 to 0.45/100,000 in 2015; the mean AFP detection rate was 0.58/100,000.

**Figure 4:**
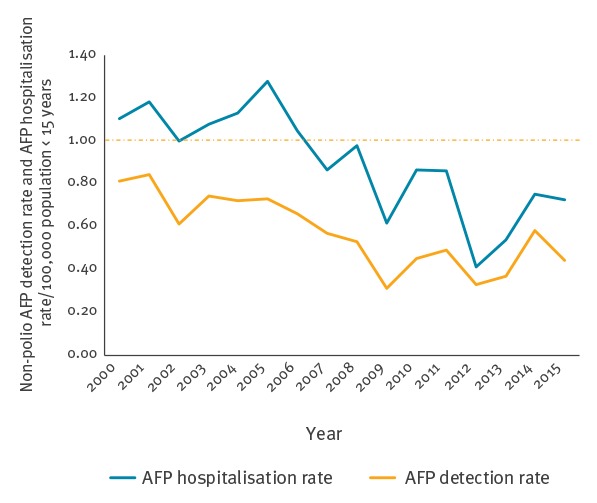
Acute flaccid paralysis (AFP) hospitalisation rate^a^ and AFP detection rate^b^ per 100,000 population under 15 years of age, Spain, 2000^c^–2015

Two periods (P) are described regarding AFP detection. The first, P1 (1998–2006), was when the AFP rate ranged from 0.78/100,000 in 1998 to 0.66/100,000 in 2006; the rate peaked in 2001 at 0.84/100,000. The second period, P2 (2007–2015), showed an AFP rate ranging from 0.57/100,000 in 2007 to 0.43/100,000 in 2015. The lowest ever reported rate was 0.31/100,000 in 2009 (Table 1; Figure 4).

AFP: acute flaccid paralysis.

^a^ Data for the AFP hospitalisation rate originated from the National Discharge Hospitalization Registry.

^b^ AFP detection rate through the AFP surveillance system.

^c^ The National Discharge Hospitalization Registry was implemented in 1998. Data from the first 2 years of implementation were of lower quality and were not included in the Figure.

Source: National Centre of Epidemiology, Instituto de Salud Carlos III and National Hospitalization Registry, Ministry of Health.

**Table 1A:** Acute flaccid paralysis (AFP) surveillance system, AFP cases expected and reported, and quality indicators of surveillance performance, Spain, 1998–2006

Parameters	Description of the parameters	WHO target	1998	1999	2000	2001	2002	2003	2004	2005	2006
**Detection and investigation**											
AFP cases reported	No further description	NA	46	40	48	51	37	45	44	44	42
AFP cases expected	Cases expected = Total population under 15 years x (1/100,000)	NA	59	59	59	61	61	61	61	60	64
Population < 15 years	No further description	NA	5,897,436	5,882,353	5,925,926	6,071,429	6,065,574	6,081,081	6,111,111	6,027,397	6,363,636
AFP detection rate	Number of AFP cases x 100,000/total population under 15 years	> 1	0.78	0.68	0.81	0.84	0.61	0.74	0.72	0.73	0.66
Reporting rate	% AFP cases with delay between paralysis onset and notification ≤ 7 days	≥ 80%	ND	55.0%	50.0%	45.0%	48.0%	43.0%	35.0%	50.0%	40.5%
Investigation rate	% AFP cases with delay between notification and investigation ≤ 2 days	≥ 80%	97.0%	97.5%	98.0%	96.0%	97.0%	93.0%	100%	100%	100%
Stool collection rate	% AFP with one faecal specimen within 14 days of paralysis onset	≥ 80%	91.3%	92.5%	97.9%	100%	94.6%	88.9%	100%	88.6%	95.2%
Adequate stool collection rate	% AFP with two faecal specimens taken ≥ 1 day apart within 14 days of paralysis onset	≥ 80%	64.0%	73.0%	69.0%	61.0%	57.0%	46.0%	56.0%	65.9%	45.2%
Surveillance index	Surveillance index = AFP detection rate up to 1.0 x adequate stool collection rate	≥ 0.8	0.50	0.50	0.56	0.51	0.35	0.34	0.50	0.48	0.30
**Zero reporting**											
Zero-reporting completeness	Annual number of AFP zero-reporting forms submitted/number of zero-reporting forms expected in the reporting year x 100	≥ 80%	ND	ND	ND	97.4%	95.6%	ND	99.8%	96.5%	98.4%
Zero-reporting timeliness	Annual number of AFP zero-reporting forms submitted by the first 7 days of the following month/number of forms expected in the reporting year x 100	≥ 80%	ND	ND	ND	73.8%	68.6%	ND	47.8%	43.9%	39.5%
**Priority cases and follow-ups**											
Hot cases	Case with a priority code (less than three doses of polio vaccine/clinical presentation compatible with polio/recent travel to endemic country/high risk group)	NA	2	1	2	6	0	2	1	1	0
Follow-up AFP rate	% AFP cases with follow-up 60 days after the date of paralysis onset	≥ 80%	100%	97.5%	100%	94.2%	92.0%	96.0%	100%	100%	100%
**Laboratory results**											
Adequate laboratory results rate	% AFP cases with laboratory results < 28 days of receiving samples at laboratory^a^	≥ 80% (1^st^)	81.4%	90.0%	68.0%	82.0%	70.0%	90.0%	81.0%	82.1%	89.5%
		≥ 80% (2^nd^)	81.1%	97.0%	67.0%	89.0%	90.0%	90.0%	86.0%	81.3%	93.8%
Non-polio enterovirus typed (NPEV) (%)	% AFP cases with positive non-polio enterovirus finding	≥ 10%	7.1%	8.1%	2.1%	9.8%	11.4%	5.0%	4.5%	2.6%	10.0%

AFP: acute flaccid paralysis; NA: not applicable; ND: not determined; WHO: World Health Organization.

^a^ Since 2015: < 14 days from receiving samples at laboratory is accepted.

Source: National Centre of Epidemiology. Instituto de Salud Carlos III.

**Table 1B:** Acute flaccid paralysis (AFP) surveillance system, AFP cases expected and reported, and quality indicators of surveillance performance, Spain, 2007–2015

Parameters	Description of the parameters	WHO target	2007	2008	2009	2010	2011	2012	2013	2014	2015
**Detection and investigation**											
AFP cases reported	No further description	NA	37	35	21	32	34	23	26	41	32
AFP cases expected	Cases expected = Total population under 15 years x (1/100,000)	NA	65	66	68	71	69	70	71	70	70
Population < 15 years	No further description	NA	6,491,228	6,603,774	6,774,194	7,111,111	6,938,776	6,969,697	7,075,637	7,046,971	7,046,971
AFP detection rate	Number AFP cases x 100,000/total population under 15 years	> 1	0.57	0.53	0.31	0.45	0.49	0.33	0.37	0.58	0.45
Reporting rate	% AFP cases with delay between paralysis onset and notification ≤ 7 days	> 80%	45.9%	40.0%	47.6%	45.2%	44.1%	52.2%	46.2%	43.9%	43.8%
Investigation rate	% AFP cases with delay between notification and investigation ≤ 2 days	> 80%	94.6%	100%	95.2%	96.8%	91.2%	95.7%	96.2%	95.1%	96.8%
Stool collection rate	% AFP with one faecal specimen within 14 days of paralysis onset	> 80%	91.9%	88.6%	85.7%	90.6%	91.2%	95.7%	92.3%	90.2%	65.6%
Adequate stool collection rate	% AFP with two faecal specimens taken ≥ 1 day apart within 14 days of paralysis onset	> 80%	56.8%	34.3%	38.1%	45.2%	38.2%	56.5%	57.7%	41.5%	46.9%
Surveillance index	Surveillance index = AFP detection rate up to 1.0 × adequate stool collection rate	> 0.8	0.32	0.18	0.12	0.20	0.19	0.18	0.21	0.24	0.21
**Zero reporting**											
Zero-reporting completeness	Annual number of AFP zero-reporting forms submitted divided by the number of zero-reporting forms expected in the reporting year x 100	> 80%	97.8%	95.6%	94.3%	97.3%	83.7%	92.0%	89.0%	99.6%	85.6%
Zero-reporting timeliness	Annual number of AFP zero-reporting forms submitted by the first 7 days of the following month divided by the number of forms expected in the reporting year x 100	> 80%	36.8%	47.8%	37.3%	37.3%	34.2%	34.2%	37.3%	41.2%	42.1%
**Priority cases and follow-up**											
Hot cases	Case with a priority code (less than three doses of polio vaccine/clinical presentation compatible with polio/recent travel to endemic country/high risk group)	NA	1	1	0	1	2	0	1	1	1
Follow-up AFP rate	% AFP cases with follow-up 60 days after the date of paralysis onset	> 80%	97.3%	100%	100%	96.8%	100%	95.7%	88.0%	95.1%	96.8%
**Laboratory results**											
Adequate laboratory results rate	% AFP cases with laboratory results <28 days of receiving samples at laboratory^a^	> 80% (1^st^ )	90.6%	81.8%	100%	92.6%	88.0%	94.4%	91.7%	88.6%	65.3%^a^
		> 80% (2^nd^ )	93.1%	91.7%	100%	86.4%	88.2%	100%	88.9%	92.3%	66.6%^a^
Non-polio enterovirus typed (NPEV) (%)	% AFP cases with positive non polio enterovirus finding	> 10%	2.9%	9.7%	0.0%	17.2%	12.9%	4.5%	4.2%	0.0%	9.5%

AFP: acute flaccid paralysis; NA: not applicable; WHO: World Health Organization.

^a^ Since 2015: <14 days from receiving samples at laboratory is accepted.

Source: National Centre of Epidemiology, Instituto de Salud Carlos III.

By age group, most cases (310, 45.7%) were under 5 years old; 29.5% (200) were between 5 and 9 years old and 24.8% (168) were between 10 and 14 years old; the youngest case was a newborn aged 16 days. Overall, a slightly higher proportion of male cases were reported (383/678, 56.4%).

Vaccination status was recorded for 659 cases (97.2%). Among them, 637 cases (96.7%) had received three or more doses of polio vaccine before being diagnosed with AFP; two cases (0.3%), both in the 5–9 year age group, were completely unvaccinated; and a further 20 cases (3.0%) had received less than three doses, including 16 under-vaccinated cases older than 6 months.

Of all AFP cases, 23 (3.4%) met hot case definition, including two VAPPs and another paralysis case produced by an iVDPV (Table 1). The risk factors associated were: clinical suspicion of poliomyelitis (3 cases), having had less than three doses of polio-vaccine (16 cases) and being immunosuppressed (9 cases); six cases had more than one risk factor concurrently.

### Clinical features

Key clinical signs suggestive of polio were collected. For cases with available information, limbs were the most common site of paralysis (535/610, 87.7%); 20.2% (130/642) had fever (≥38°C) at paralysis onset; 40.4% (227/562) presented rapid progression to complete paralysis and 8.2% (50/610) had asymmetric paralysis.

All the AFP cases were hospitalised, mainly in highly specialised tertiary hospitals. For 655 (96.6%) of them a clinical diagnosis was reached. Among them, Guillain–Barré syndrome (GBS), including Miller Fisher syndrome and other polyradiculoneuritis, was the most common clinical diagnosis in each year of the whole study period (504, 76.9%), followed by myelitis (44 cases, 6.7%) and other uncommon diagnoses like encephalomyelitis or meningitis (23 cases, 3.5%). Other rare reported diagnoses were tumours or multiple sclerosis.

The information on follow-up was collected in 651 cases (96.0%), of which 179 (27.4%) presented residual paralysis after 60 days of paralysis onset.

Most cases were classified as non-polio AFP (675, 99.0%), except three cases who occurred due to a Sabin-like poliovirus (PV-SL) or an iVDPV. In 1999 a 5-month-old infant presented with rapidly evolving asymmetric paralysis, having received the first OPV dose one month before; a PV-SL3 was detected in stool samples. The infant recovered but residual paralysis persisted. In 2001, a 7-month-old infant, having received the second dose of OPV 3 months before, presented with asymmetric paralysis with a rapid evolution and PV-SL2 was identified in stool samples. Concurrent severe immunosuppression was diagnosed and the child died.

Later, in 2005, an AFP case imported from Morocco and resulting from a recombinant iVDPV type 2 was found. The case was a 14-month-old boy with residual paralysis and major histocompatibility complex Class II immunodeficiency; he had received two OPV doses, the second at 6 months of age. Eight months later the patient developed encephalitis with clinical tetraparexia, at which point suspicion of polio was raised and diagnosis confirmed. The high level of vaccination coverage in Spain and the rapid tracing and monitoring of close contacts prevented virus spread [15].

### Laboratory investigations

For 92.3% (626) of the total AFP reported cases, at least one stool sample was collected. Taking into account that several samples from the same person could be collected, from 1998 to 2004, 14 stool samples taken from AFP cases or from their contacts were positive for PV-SL. A PV-SL3 and a PV-SL2 were isolated from two VAPP cases. In addition, during 2005, an iVDPV type 2 was detected (Table 2). Furthermore, the NPL performs the characterisation of all NPEVs isolated. EV species B were the most frequent serotypes found in the stool samples (30; 4.4% of AFP cases), although several Coxsackievirus A (from species A) were also detected. An EV-D68 was identified in stools from a 4 year-old child with severe limb paralysis in 2015 (Table 2).

**Table 2:** Poliovirus and non-polio enterovirus detected in stool samples from reported acute flaccid paralysis cases, Spain, 1998–2015

Cases of AFP and enteroviruses detected	1998	1999	2000	2001	2002		2003	2004	2005	2006	2007	2008	2009	2010	2011	2012	2013	2014	2015	Total
Number of reported AFP cases	46	40	48	51	37		45	44	44	42	37	35	21	32	34	23	26	41	32	**678**
PV isolated	0	PV-SL^a^	0	PV-SL^b^	0		0	0	iVDPV-2^c^	0	0	0	0	0	0	0	0	0	0	**3**
**NPEV serotypes**																				
Echovirus 3	2	0	0	0		1	0	0	0	0	0	1	0	0	0	0	1	0	0	**5**
Echovirus 6	0	0	0	0		0	0	0	0	1	0	1	0	0	0	1	0	0	0	**3**
Echovirus 7	1	0	0	0		1	0	0	0	0	0	0	0	3	0	0	0	0	0	**5**
Echovirus 9	0	0	0	0		0	0	0	0	1	0	0	0	0	1	0	0	0	0	**2**
Echovirus 11	0	2	0	0		0	0	0	0	0	0	0	0	0	1	0	0	0	0	**3**
Echovirus 13	0	0	1	0		0	0	1	0	0	0	0	0	0	0	0	0	0	0	**2**
Echovirus 14	0	0	0	0		0	0	0	0	1	0	0	0	0	0	0	0	0	0	**1**
Echovirus 18	0	0	0	1		0	0	0	0	0	0	0	0	0	0	0	0	0	0	**1**
Echovirus 21	0	0	0	0		0	1	0	0	0	0	0	0	0	0	0	0	0	0	**1**
Echovirus 20	0	0	0	0		1	0	0	0	0	0	0	0	0	0	0	0	0	0	**1**
Echovirus 25	0	0	0	0		0	0	0	0	0	0	0	0	0	1	0	0	0	0	**1**
Echovirus 30	0	0	0	1		0	0	1	0	0	0	1	0	0	1	0	0	0	0	**4**
Echovirus 33	0	0	0	0		0	0	0	0	1	0	0	0	0	0	0	0	0	0	**1**
Coxsackievirus A9	0	1	0	0		0	0	0	0	0	0	0	0	0	0	0	0	0	0	**1**
Coxsackievirus B2	0	0	0	1		0	0	0	0	0	0	0	0	0	0	0	0	0	0	**1**
Coxsackievirus A16	0	0	0	0		1	0	0	0	0	0	0	0	1	0	0	0	0	0	**2**
Coxsackievirus A22	0	0	0	0		0	0	0	0	0	0	0	0	0	0	0	0	0	1	**1**
Coxsackievirus A24	0	0	0	0		0	0	0	1	0	0	0	0	0	0	0	0	0	0	**1**
Enterovirus C-99	0	0	0	0		0	0	0	0	0	0	0	0	1	0	0	0	0	0	**1**
Enterovirus D-68	0	0	0	0		0	0	0	0	0	0	0	0	0	0	0	0	0	1	**1**
Non-typed EV	0	0	0	2		0	1	0	0	0	1	0	0	0	0	0	0	0	0	**4**
**Total of AFP cases with isolated NPEV**	**3**	**3**	**1**	**5**		**4**	**2**	**2**	**1**	**4**	**1**	**3**	**0**	**5**	**4**	**1**	**1**	**0**	**2**	**42**

AFP: acute flaccid paralysis; EV: enterovirus; iVDPV: immunocompromised vaccine-derived poliovirus; NPEV: non-polio enterovirus; PV: poliovirus; PV-SL: Sabin-like poliovirus; VAPP: vaccine-associated paralytic poliomyelitis.

^a^ 1 VAPP-PVSL3.

^b^ 1 VAPP-PVSL2.

^c^ Imported case.

Source: National Poliovirus Laboratory and National Centre of Epidemiology, Instituto de Salud Carlos III.

### Quality indicators of acute flaccid paralysis surveillance performance

The quality of AFP surveillance has weakened over the surveillance system’s lifespan. The sensitivity of the surveillance, the AFP detection rate, has gradually decreased from the WHO-stated objective of at least one case per 100,000 inhabitants under 15 years of age (Table 1; Figure 4). The completeness of zero reporting has always been maintained at over 80%, while the timeliness of zero reporting decreased from 73.8% in 2001 to 34.2% in 2012 (Table 1). The number of hospitalisations with an AFP diagnosis identified in hospital discharge registry searches shows a fluctuating pattern. A variable gap is described between the AFP hospitalisation rate and the AFP detection rate across the study period (Figure 4).

Timeliness of investigation (i.e. the investigation rate) reached the 80% target every year and for most cases at least one faecal specimen was taken; however, the ‘adequate stool collection rate‘ always remained under the expected 80%, and seemed to decrease slightly during the second period (2007–2015) compared with the first period (1998–2006). Consequently, the ‘surveillance index’ experienced a decrease from 0.50 in 1998 to 0.21 in 2015 (Table 1).

The ‘adequate laboratory results rate’ has been always above 80% except for 2015, when the new diagnosis policy began and the laboratory results had to be communicated sooner (14 days instead of 28 days). After two-year period of adaptation, laboratory capabilities recovered; for 2017 the ‘adequate laboratory results rate’ exceeded the quality target required (82.4% for the first sample and 85.7% for the second one) [16]. During the study period, the proportion of AFP cases with positive NPEV findings ranged from 17.2% in 2010 to 0% in 2009 and 2014 (Table 1).

## Discussion

In Spain the last endemic case produced by a WPV occurred in 1988 in an under-immunised population. After that event Spain progressively improved the polio vaccination coverage (since 1996 national coverage of three doses exceeds 95%) allowing to reach a high population immunity against polio. Three additional imported cases associated with WPV were reported between 1980 and 1989. Since 2004 OPV is no longer administered in Spain and the last cases associated with PV-SL were reported in 1999 and 2001 [8,17-19]. The European Regional Certification Commission for Poliomyelitis Eradication classifies Spain as at low risk of WPV transmission. The maintenance of high polio vaccination coverage minimises the risk of spread in case of importation, and a good-quality surveillance system ensures the timely poliovirus detection [6].

The quality of AFP surveillance decreased with time, particularly after the OPV to IPV switch, reflecting the loss of awareness about polio, when finding cases of paralysis produced by WPV or associated to vaccine virus are very unlikely.

During the studied period, the number of AFP cases reported underwent similar fluctuations to the rate of children hospitalised with a clinical compatible AFP diagnosis; the fact that the hospitalisations and notifications follow the same pattern, indicates that the surveillance is working relatively well despite it detecting less than one case of AFP per 100,000.

In our experience, the WHO’s expected rate of at least one case of AFP per 100,000 population under 15 years of age could be overestimating the real rate of this syndrome among children in a country like Spain. 

Although active feedback has been proven not to increase the proportion of reported and virologically investigated patients [20], in our experience zero reporting acts as a monthly reminder maintaining awareness about polio among clinicians who tend to believe it is a long-disappeared disease. It also ensures that the entire country takes part in the surveillance and the whole population is represented. In Spain, zero cases are reported, but the delay in notification is increasingly evident.

About half of reported AFP cases are notified more than a week after paralysis onset. Low concern levels regarding polio and the delay in consulting for symptoms of insidious onset, such as some type of paralysis, are the main causes of this reporting delay and may minimise the chance to implement control measures and/or appropriate sample collection. This is reflected by the ‘adequate stool collection rate’, which did not reach the expected 80% over the study period and the decrease of the ‘surveillance index’ during that time. Nevertheless, the investigation rate every year reaches the 80% target, showing that once a case enters the system, the investigation is conducted promptly.

In addition to discarding poliovirus infection, AFP laboratory diagnosis can lead to identifying other EVs in patients’ stool samples. In fact, the proportion of AFP cases with positive NPEV findings is used as an indicator for the quality of the laboratory’s EV typing.

The most frequent EVs identified in the samples from AFP cases across the study period were EVs from species B (echovirus 3, echovirus 7 and echovirus 30); similar results were found in laboratory EV surveillance [17]. A systematic review about EV detections in cases with AFP showed that in surveillance studies, EVs from species B were among the most frequently detected [21].

The recent increase in the detection of EVs associated with severe neurological symptoms in children across Europe [22,23] has also been identified in Spain. Of particular concern was a an EV-A71 outbreak notified in 2016 with many severe encephalitis cases in paediatric patients [24,25].

The first case of paediatric neurological disease associated to EV-D68 infection in Spain was reported to the national AFP surveillance system late in 2015 [16]. During 2016, two additional AFP cases associated with EV-D68 [26] and 12 AFP cases associated with EV-A71 were reported highlighting the importance of maintaining AFP surveillance to detect concerning signals of unexpected EV circulation in a country [16]. Any surveillance system devoted to monitoring the spread of EVs should take advantage of existing networks, such as AFP surveillance systems, non-polio EV laboratory networks or viral meningitis surveillance [22,27,28].

In a polio-free IPV-user country, poliomyelitis can arise and spread to contacts who are not properly vaccinated. Movements of people from areas still using OPV, under-vaccinated population groups, foreign-born children coming from endemic countries or hard-to-reach communities can lead to the identification of AFP hot cases, and will trigger the Standard Operational Procedure until the case can be discarded [9]. The aim of the response plan is to implement activities aimed at interrupting any poliovirus transmission within 120 days of confirmation. The response activities will be promptly implemented when a suspected case of polio or priority AFP case is reported, or when a poliovirus is identified by the EV surveillance or by the environmental surveillance system [9].

Re-emergence still may arise from prolonged asymptomatic excretion of poliovirus by hospitalised primary immune deficient (PID) patients, through repeated exposure of close contacts. PID patients with a recent OPV vaccination should be identified and screened for any poliovirus excretions [29].

In 2017 no WPV transmission in the WHO European Region occurred [6]. One of the milestones achieved in 2016 was the successful switch from trivalent to bivalent polio vaccine by all countries in the Region that still used OPV [30]. Cessation of the use of OPV is necessary to eliminate the low long-term risks of VDPVs associated with its use. Countries with IPV in their vaccination schedule commonly share borders with OPV-user countries, and residents may travel back and forth, increasing the risk of VDPVs’ circulation.

The quality of AFP surveillance in the WHO European Region as a whole has not declined in recent years, but there are indications that vaccine coverage is in decline in a small number of countries, which is of concern. In particular, Bosnia and Herzegovina, Romania and Ukraine were considered to be at high risk of a sustained polio outbreak due to suboptimal performance of poliovirus surveillance and the low population immunity [6]. A further 25 countries have been considered as having an intermediate risk and an additional 24 as having a low risk of sustained transmission in the event of WPV importation or emergence of VDPV [6].

Surveillance of AFP is considered as a milestone and the most efficient form of surveillance in the last phase of polio eradication. Maintaining clinical surveillance of poliomyelitis in a polio-free territory is hard. Poliomyelitis is among 67 other diseases under mandatory notification to the Spanish National Surveillance System. Reviewing the history of AFP surveillance in Spain between 1998 and 2015 brings up the need for awareness by all healthcare professionals involved: medical staff in charge of paediatrics, child neurology or paediatric intensive care units and the personnel from the laboratories involved in diagnosis of these patients’ clinical samples.

AFP surveillance along with non-polio EV laboratory surveillance must be maintained and enhanced both to help polio eradication and to establish a sensitive surveillance for prompt detection of emergent and unexpected circulation of other EVs.

## Notes

**Members of the Spanish AFP Surveillance Working Group including Laboratory Network:** Andalucía: S. Sanbonmatsu, JM. Navarro (Laboratorio del Hospital Universitario Virgen de las Nieves, Granada, Spain); Aragón: M. Omeñaca (Laboratorio del Hospital Universitario Miguel Servet, Zaragoza, Spain); Canarias: C. Pérez-González (Laboratorio del Hospital Universitario Dr Negrín, Las Palmas de Gran Canarias, Spain); Cataluña: M del Cuerpo (Laboratorio de Microbiología. Hospital Santa Creu i Sant Pau, Barcelona, Spain); C. Valenciana: S. Guiral (Servei de Vigilància i Control Epidemiològic. Conselleria de Sanitat Universal i Salut Pública. Comunitat Valenciana. Spain); Extremadura: JM. Ramos (Subdirección de Epidemiología. D.G. de Salud Pública. Consejería de Salud y Políticas Sociales. Extremadura, Spain); Murcia: A. García-Fulgueiras (Servicio de Epidemiología. D. G. de Salud Pública y Adicciones. Consejería de Salud. Región de Murcia, Spain); Navarra: A. Barricarte (Servicio de Epidemiología y Prevención Sanitaria. Instituto de Salud Pública de Navarra. CIBERESP Spain); La Rioja: E. Martínez Ochoa (Sección de Vigilancia Epidemiológica y Control de Enfermedades Transmisibles, D.G de Salud Pública y Consumo, Consejería de Salud, La Rioja, Spain).
